# Green Sturgeon Physical Habitat Use in the Coastal Pacific Ocean

**DOI:** 10.1371/journal.pone.0025156

**Published:** 2011-09-22

**Authors:** David D. Huff, Steven T. Lindley, Polly S. Rankin, Ethan A. Mora

**Affiliations:** 1 National Marine Fisheries Service, National Oceanic and Atmospheric Administration, Santa Cruz, California, United States of America; 2 Oregon Department of Fish and Wildlife, Newport, Oregon, United States of America; 3 Department of Wildlife, Fish and Conservation Biology, University of California Davis, Davis, California, United States of America; University of Canterbury, New Zealand

## Abstract

The green sturgeon (*Acipenser medirostris*) is a highly migratory, oceanic, anadromous species with a complex life history that makes it vulnerable to species-wide threats in both freshwater and at sea. Green sturgeon population declines have preceded legal protection and curtailment of activities in marine environments deemed to increase its extinction risk. Yet, its marine habitat is poorly understood. We built a statistical model to characterize green sturgeon marine habitat using data from a coastal tracking array located along the Siletz Reef near Newport, Oregon, USA that recorded the passage of 37 acoustically tagged green sturgeon. We classified seafloor physical habitat features with high-resolution bathymetric and backscatter data. We then described the distribution of habitat components and their relationship to green sturgeon presence using ordination and subsequently used generalized linear model selection to identify important habitat components. Finally, we summarized depth and temperature recordings from seven green sturgeon present off the Oregon coast that were fitted with pop-off archival geolocation tags. Our analyses indicated that green sturgeon, on average, spent a longer duration in areas with high seafloor complexity, especially where a greater proportion of the substrate consists of boulders. Green sturgeon in marine habitats are primarily found at depths of 20–60 meters and from 9.5–16.0°C. Many sturgeon in this study were likely migrating in a northward direction, moving deeper, and may have been using complex seafloor habitat because it coincides with the distribution of benthic prey taxa or provides refuge from predators. Identifying important green sturgeon marine habitat is an essential step towards accurately defining the conditions that are necessary for its survival and will eventually yield range-wide, spatially explicit predictions of green sturgeon distribution.

## Introduction

The green sturgeon's (*Acipenser medirostris*) complex life history causes it to be vulnerable to numerous threats in both freshwater and at sea [1], [2]. It is a highly oceanic and migratory, anadromous species [3], [4] that is captured as bycatch in white sturgeon commercial and sport fisheries, tribal salmon gillnet fisheries, and coastal groundfish trawl fisheries [1]. Population declines have preceded legal protection and subsequent curtailment of activities deemed to increase extinction risk [1], [5]. Yet, green sturgeon coastal habitat is poorly understood [4], [6], [7]. At present, the marine habitats of oceanic anadromous sturgeon species have been characterized only generally, and specific information regarding marine habitat associations of green sturgeon is almost totally lacking [8].

Inadequate knowledge of green sturgeon ecology may necessitate overly restrictive measures by fishery managers who must choose more conservative options for protection in the absence of specific information regarding habitat requirements. For green sturgeon, a lack of knowledge may be especially problematic because it is the most widely distributed and marine oriented member of the sturgeon family [3]; consequently, unnecessarily encumbering regulations could have broad-scale effects. Currently, 30,890 km of coastal marine habitat extending to the 110 m isobath along the West Coast of the United States has been designated as “critical” for green sturgeon under the United States Endangered Species Act (ESA) [5]. Improved knowledge of green sturgeon habitat within these waters could lead to more geographically or temporally specific protection.

Although green sturgeon use various environments throughout their life cycle, they spend most of their lives in the coastal ocean [3]. Green sturgeon generally spend their first two years in freshwater rivers before they migrate to marine habitats [6], [9]. At about age 15, they return to their natal rivers to spawn in the spring, and depart for marine waters the following autumn. They will continue to spawn every 2–4 years afterwards [10], [11], [12]. Subadults and adults also commonly visit bays and estuaries during summer and early autumn [13], [14]. Recent investigations have elucidated the ocean distribution of green sturgeon and have revealed some remarkable migration and aggregation behaviors during ocean residence [4], [6]. They remain in relatively shallow depths (40–100 m) and may travel long distances (>40 km/day, up to 1000 km) that include northward migrations in the winter followed by southward migrations in the summer [4], [8]. Green sturgeon from different natal rivers also congregate in great numbers at specific sites, presumably to exploit superior foraging opportunities [4]. Recurrent concentrations of sturgeon in nearshore zones indicate that suitable areas are likely limited in number [6].

Characterizing sturgeon habitat in marine environments presents unique challenges. In order to quantify habitat associations, sturgeon must be observed directly, captured, or monitored with electronic devices such as pop-off archival geolocation tags (PATs) and acoustic tags. Direct observation is not feasible because sturgeon are relatively rare, occur at great depths, and swim too rapidly for SCUBA divers or remotely operated vehicles to follow, especially in low light or turbid conditions. Habitat associations have formerly been inferred from bottom trawl capture records [6], [15], [16], but bottom trawls are inappropriate for delineating the full range habitat use because they do not perform well on complex bottom topography, or where the slope is very steep [17]. The use of gill nets in combination with non-electronic tags is feasible, but this usually requires large sample sizes because the majority of tagged fish are never recaptured, and is therefore not practical for rare species such as green sturgeon. Furthermore, non-electronic tags provide little information regarding the duration of residence in a specific location. Electronic devices such as pop-off archival tags (PATs) and acoustic tags have proven useful for habitat studies of marine fishes [18], [19], but success has been limited with sturgeon in marine habitats [6], [7]. The primary restrictive factors for PATs have been cost, which usually limits the sample size [6], and difficulties in calculating day length or time of zenith caused by complex topography, turbidity, or other factors associated with deep, benthic habitats that may interfere with light detection [20]. Alternatively, acoustic tags that emit an ultrasonic signal may prove to be a viable option for delineating nearshore benthic marine habitats, especially when used in conjunction with multiple stationary data logging hydrophones [19], [21], [22], [23].

**Figure 1:**
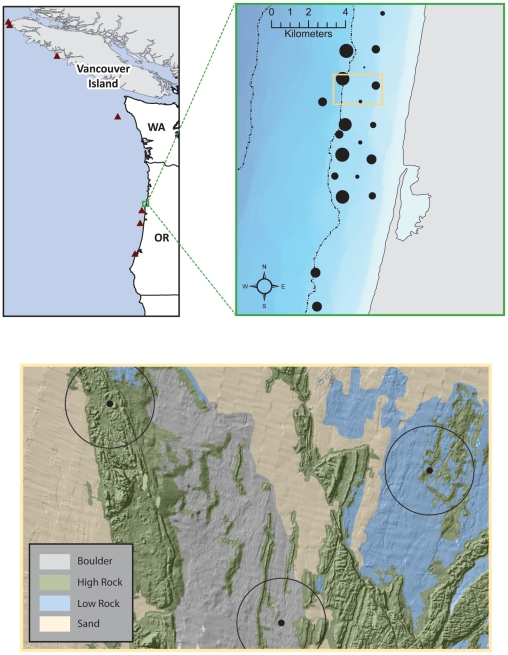
Map of the study area. *Top-left inset*: Overview of Oregon and Washington, USA coast with hydroacoustic receiver array area outlined in green. Red triangles indicate PAT detachment locations. *Top-right inset*: overview of hydroacoustic receiver array with black dots that represent individual hydrophones sized proportionally so that larger dots indicate greater duration of green sturgeon presence (min = 1, max = 19, mean = 8.2 days per station). Dotted lines indicate 40 m bathymetric depth contours. The tan-colored outline box indicates the area shown in the bottom inset. *Bottom inset*: detailed view of a portion of the study area with hill shaded bottom topography overlaid with substrate type. Circumscribed black dots indicate hydrophone locations with 250 m buffers.

Coastal tracking arrays designed as acoustic “grids” or “gates” that consist of numerous hydrophones arranged in patterns to record the passage of acoustically tagged animals have been deployed on the continental shelf of western North America [24], [25]. Our objective was to characterize green sturgeon habitat using detection data collected in 2006 for 37 acoustically tagged green sturgeon from one such array, located along the Siletz Reef near Newport, Oregon (Figure 1). The Siletz Reef has a highly variable bathymetry and variable bottom topography. We classified seafloor physical habitat features with broad-scale, high-resolution bathymetric and backscatter data from sonar survey of the Siletz Reef [26], [27]. We then described the distribution of selected habitat components and their relationship to green sturgeon presence across the study area with a multivariate analysis, non-metric multidimensional scaling (NMS). We also constructed generalized linear models (GLMs) to describe the mean response of sturgeon in our study to physical habitat features, identify habitat components that are important to green sturgeon, and compare and corroborate patterns detected in the multivariate analysis. Finally, we summarized data from eight green sturgeon tagged with PATs and released near the Oregon coast in 2004. We used these data to confirm previous studies describing depth and water temperatures inhabited by green sturgeon, in addition to the timing of movements from bays and estuaries to oceanic habitats [6], [11], [28].

## Results

**Figure 2:**
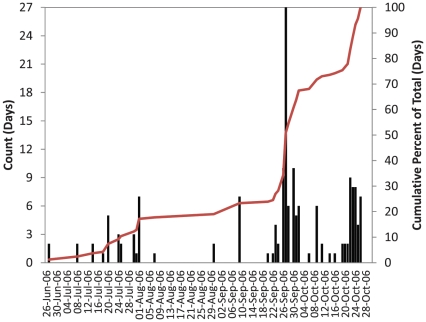
Histogram and Cumulative Percentage Plots for Acoustic Tag Detections. Frequency (left vertical axis) and cumulative percentage (right vertical axis) of detection-days by date for 37 sturgeon detected among 20 hydroacoustic receivers used in this analysis.

We detected thirty-seven green sturgeon (Table 1) at twenty hydrophones where reclassified side-scan sonar data and high-resolution bathymetry data were available, for a sum of 163 detection-days. Sturgeon detected in the hydrophone array were originally tagged and released in various locations from San Pablo Bay, California in the south to Grays Harbor, Washington in the north (Table 1). We recorded relatively fewer detection-days (46 d, 28%) within the receiver array area from 27 June to 24 September 2006; the remaining detection-days (117 d, 72%) occurred from 26 September to 26 October 2006 (Figure 2). The mean number of detection-days per fish was 4.4 (Min = 1, Max = 12, SD = 3.5) and each sturgeon visited a mean of 4 (Min = 1, Max = 11, SD = 3) hydrophones. Six sturgeon visited a given hydrophone for more than one day; four of these fish spent two days at a single hydrophone and two sturgeon spent from one to three days at a single hydrophone.

**Table 1:** Total length, release date, and release locations for 37 acoustically tagged sturgeon.

Total Length (cm)	Tag Type/Serial Number	Release Date	Release Location	Release Latitude	Release Longitude
178	A/6724F	3-Aug-2005	Grays Harbor	46.9582	−123.9967
175	A/6716F	21-Jul-2005	Grays Harbor	46.9578	−123.9950
156	A/6759F	7-Sep-2005	Grays Harbor	46.9570	−123.9982
160	A/6762F	6-Sep-2005	Grays Harbor	46.9568	−123.9958
152	A/6733F	1-Sep-2005	Grays Harbor	46.9558	−123.9973
188	A/6735F	1-Sep-2005	Grays Harbor	46.9558	−123.9973
147	A/5309D	29-Aug-2003	Willapa Bay	46.5194	−124.0027
195	A/5300D	20-Aug-2003	Willapa Bay	46.5188	−124.0011
132	A/1633F	29-Jun-2004	Willapa Bay	46.5184	−124.0056
147	A/5716D	27-Aug-2003	Willapa Bay	46.5171	−124.0034
152	A/5696D	29-Aug-2003	Willapa Bay	46.5169	−124.0053
139	A/1638F	30-Jun-2004	Willapa Bay	46.5124	−124.0117
174	A/5711D	5-Sep-2003	Willapa Bay	46.5075	−124.0066
160	A/5700D	2-Sep-2003	Willapa Bay	46.5063	−124.0065
177	A/5727D	10-Jun-2004	Willapa Bay	46.3077	−124.0042
195	PAT/43031	11-Aug-2004	Columbia River	46.2458	−123.7565
222	PAT/43030	11-Aug-2004	Columbia River	46.2451	−123.7625
166	PAT/43033	11-Aug-2004	Columbia River	46.2451	−123.7625
180	PAT/43032	22-Jul-2004	Columbia River	46.2374	−123.7378
171	PAT/43035	22-Jul-2004	Columbia River	46.2374	−123.7378
167	A/1639F	14-Jul-2004	Columbia River	46.2369	−123.7371
210	A/5664F	10-Sep-2004	Columbia River	46.2360	−123.7375
158	PAT/43029	23-Jul-2004	Columbia River	46.2354	−123.7367
196	PAT/43027	10-Aug-2004	Columbia River	46.2350	−123.7369
213	A/5660D	6-Oct-2003	Rogue River	42.5610	−124.0970
163	A/5662D	6-Oct-2003	Rogue River	42.5610	−124.0970
179	A/5669D	7-Oct-2003	Rogue River	42.5610	−124.0970
150	A/5690D	9-Oct-2003	Rogue River	42.5270	−124.1560
207	PAT/43034	11-Aug-2004	Rogue River	42.4304	−124.4001
203	A/1069G	15-Jul-2005	Sacramento River	39.7550	−122.0291
193	A/1066G	1-Aug-2005	Sacramento River	39.7550	−122.0291
193	A/1073G	3-Aug-2005	Sacramento River	39.7550	−122.0291
193	A/1064G	4-Aug-2005	Sacramento River	39.7550	−122.0291
168	A/1065G	4-Aug-2005	Sacramento River	39.7550	−122.0291
203	A/1072G	4-Aug-2005	Sacramento River	39.7550	−122.0291
203	A/1088G	8-Sep-2005	Sacramento River	39.7550	−122.0291
208	A/1090G	17-Sep-2005	Sacramento River	39.7550	−122.0291
173	A/1081G	29-Sep-2005	Sacramento River	39.7550	−122.0291
198	A/1062G	1-Oct-2005	Sacramento River	39.7550	−122.0291
183	A/1089G	19-Oct-2005	Sacramento River	39.7550	−122.0291
163	A/5100G	22-Oct-2005	Sacramento River	39.7550	−122.0291
162	A/9170D	10-Aug-2004	San Pablo Bay	38.0500	−122.3833
137	A/5262F	24-Aug-2004	San Pablo Bay	38.0500	−122.3833
204	A/7999F	1-Sep-2005	San Pablo Bay	38.0500	−122.3833
140	A/8009F	6-Sep-2005	San Pablo Bay	38.0500	−122.3833

Tag type: A = acoustic tag (Mean length = 174 cm, Min = 132 cm, Max = 213 cm), PAT = pop-off satellite tag (Mean length = 187 cm, Min = 158 cm, Max = 222 cm).

There was no observable bias in the spatial distribution of sturgeon presence within the hydrophone array without reference to bottom type; except that a greater number of detection-days tended to occur along the north to south, 40 m depth contour (Figure 1). Visual inspection of habitat maps (e.g. Figure 1, bottom inset) and quantification of habitat components (Table 2) confirmed that hydrophone buffers included a high degree of habitat heterogeneity representative of the study area. Of the substrate types in the receiver buffer areas, sand (40%) and low relief rock (38%) occurred in the greatest proportions, while boulders (5%) and high relief rock (12%) were the least prevalent. For benthic position index (BPI) categories, upper slope habitat (3%) was the least abundant, while flat/plain habitat (36%) formed the greatest percentage. The remaining BPI categories occurred in roughly equivalent proportions (∼10%). The study area had a predominantly west/southwest aspect consistent with westerly ocean depth increase from the coastline (Figure 1). Rugosity values indicated, on average, a low to medium degree of relief in the hydrophone buffer zones [29].

**Table 2:** Mean and standard deviation for habitat variables within hydrophone buffers and maximum linear and contour (surface) correlations (R^2^) of the habitat variables with the NMS ordination scores.

Variable	Mean	SD	± w/NMS	Linear R^2^	Surface R^2^
Depth (m)	19.7	5.01	negative	0.443	0.576
Rugosity	1.02	0.014	positive	0.641	0.314
Sand	40%	25%	negative	0.644	0.206
Low Relief Rock	38%	24%	positive	0.014	0.473
High Relief Rock	12%	21%	positive	0.587	0.790
Boulders	5%	13%	positive	0.028	0.095
Valley/Crevice	10%	12%	positive	0.000	0.466
Lower Slope	8%	7%	positive	0.220	0.021
Flat/Plain	36%	24%	positive	0.525	0.382
Middle Slope	11%	8%	positive	0.498	0.396
Upper Slope	3%	3%	positive	0.629	0.359
Peak/Ridge	10%	10%	positive	0.512	0.733
Aspect	West	n/a	negative	0.233	0.016

Positive or negative relationship with NMS (± w/NMS) describes the linear correlation of horizontal axis scores with sturgeon detection-days at each hydrophone. The mode of aspect values is shown instead of the mean (West, n = 13; Southwest, n = 7).

We described the relationship of individual sturgeon encountered in our study to habitat features by positioning them on a non-metric multidimensional scaling (NMS) plot according to covariation and association among the cumulative duration of presence within the hydrophone areas. We chose a three dimensional solution for the NMS ordination by examining scree plots, and after Monte Carlo runs obtained a p-value = 0.004, suggesting that the final stress value had a low probability of occurring by chance [30]. Stress for the final solution after 122 iterations was 15. The proportion of variance represented by three axes between the original distance matrix and the ordination distances was R^2^ = 0.814.

**Figure 3:**
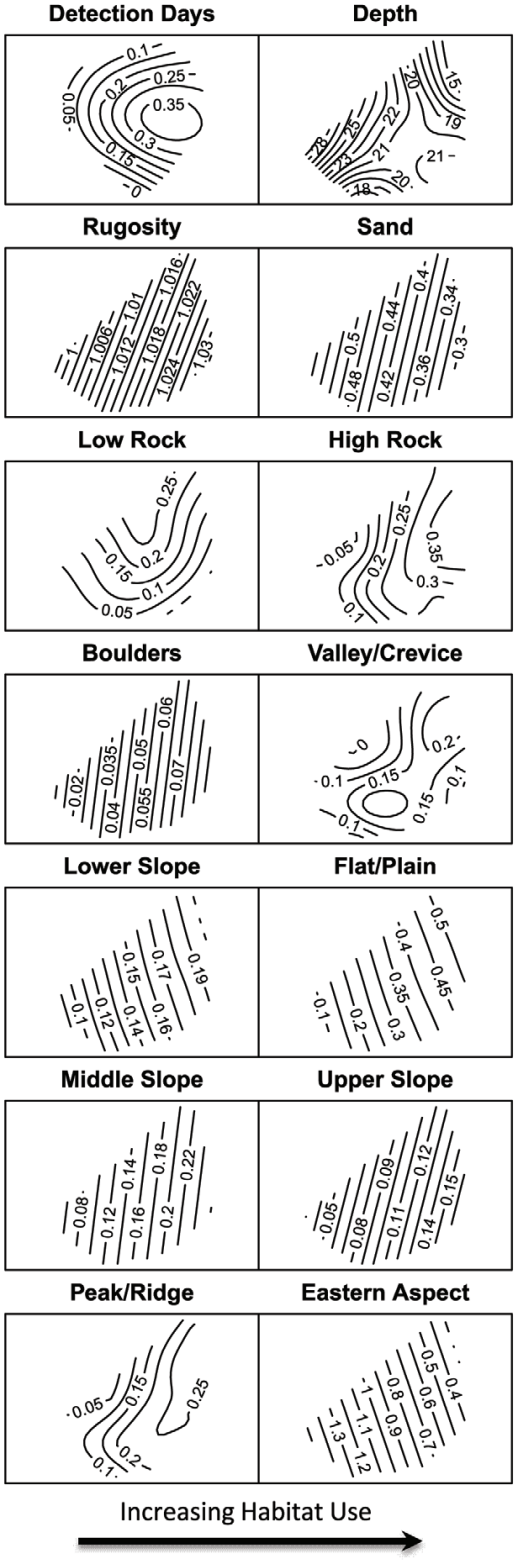
Ordination Plots. Non-metric multidimensional scaling (NMS) ordination with fitted regression surfaces that describe the responses of green sturgeon hydrophone detections to habitat variables in the Siletz Reef array. The isolines represent the predicted smooth trends by general additive model (GAM) between environmental variables and plot scores. We rotated NMS plots to maximize the linear correlation of sturgeon detection-days with NMS horizontal axis (axis 1) scores from left to right. Sturgeon presence at the hydrophones therefore tends to increase from left to right along the horizontal axis. Negligible variance in sturgeon presence is represented on the vertical axis (axis 2). We omitted NMS plot score points to improve clarity.

In general, the NMS analysis demonstrated that habitat components associated with greater seafloor complexity were positively related to sturgeon presence, whereas one component, proportion of sand, which exemplified reduced seafloor complexity, was negatively associated with sturgeon presence. Our indirect gradient analysis using NMS-fitted contour plots identified both strong linear and non-linear relationships between the green sturgeon presence gradient and different habitat components (Figure 3). Rotating the NMS plot to maximize the linear correlation of sturgeon detection-days with NMS (horizontal) Axis 1 scores resulted in a coefficient of determination (R^2^) of 0.392, and negligible variance in sturgeon detection-days was represented on the other two axes. Habitat component gradients represented on axes 2 and 3 therefore have a negligible linear relationship to green sturgeon detection-days. Higher R^2^ for fitted contours (non-linear) than for horizontal axis scores (linear) indicated a strong (R^2^>0.5) non-linear response of sturgeon detection-days (fitted surface R^2^ = 0.609) depth, high relief rock, low relief rock, valley/crevice and peak/ridge (R^2^ values in Table 2). Duration of sturgeon presence, characterized by the non-linear surface in the top-left contour plot in Figure 3, generally increased from left to right. All of the habitat components except three (depth, eastern aspect, and sand) exhibited an increasing relationship (both linear and non-linear) with the horizontal axis scores from left to right, and therefore duration of sturgeon presence. The strongest positive relationship with the horizontal axis by linear correlation was rugosity (R^2^ = 0.641), which represented structural complexity. The strongest non-linear positive relationship was the proportion high relief rock (R^2^ = 0.790), which is defined as hard surface with a greater than 45° angle. The strongest negative linear relationship with the horizontal axis, was the proportion of sand (R^2^ = 0.644), whereas the strongest negative non-linear variable was depth (R^2^ = 0.576). The BPI categories all had positive relationships with the horizontal axis, the strongest of these was the proportion of habitat that are upper slopes (linear R^2^ = 0.629), also known as escarpments [29], and peak/ridges (non-linear R^2^ = 0.733) which are simply high points in the terrain.

**Figure 4:**
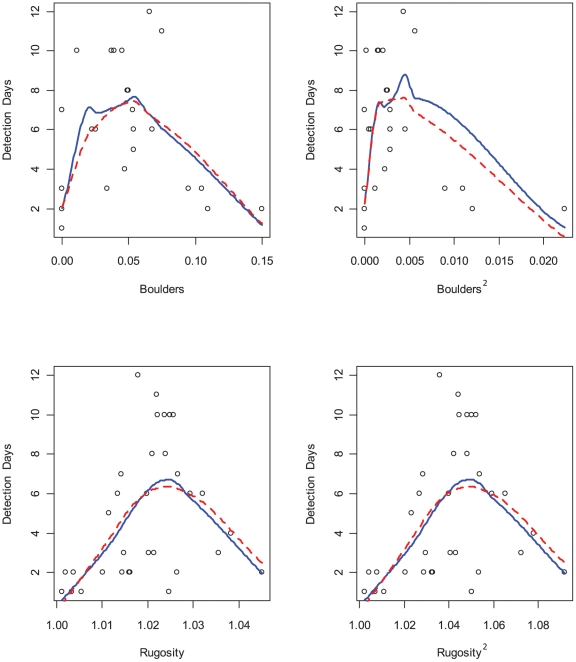
Marginal Model Plots. Four marginal model plots for green sturgeon detection data showing the response variable (Detection-Days) on the vertical axes and the horizontal axes denote numeric predictor values (plotted points) in the final GLM model. Margin model plots provide a graphical representation of model fit by showing the marginal relationships between the response and each predictor. We fitted a regression function for each of the plots using a lowess smooth function for the data (solid blue line) and for the fitted values (dashed red line).

Our underlying motive for constructing and selecting GLMs was to identify habitat components that are of biological importance to green sturgeon and produce a model that may be used to identify likely sturgeon habitat at some point in the future. Of the 5036 models evaluated using qAIC values, green sturgeon detection-days was best explained by a GLM of the following form (Table 3):
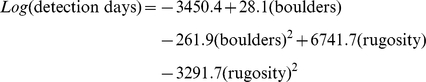
This model received far more support than any other model; it ranked first with the lowest qAIC value and was attributed nearly all of the Akaike weight (*w* = 0.9996), indicating that it was very likely the best model for the observed data, given the candidate set of models [31]. With clear support for this single model, it was unnecessary to consider model averaging to reduce model selection bias and uncertainty [32]. Each of the terms in the model led to significant reductions in model deviance, except Rugosity^2^, which was only marginally significant, and the null model deviance was reduced from 96.0 to 21.9 in the final model (Table 3). The cross-validated R^2^ for this model, generated by leave-one-out jackknifing, was 0.73 and the jackknifed root mean squared error was 3.95 detection-days. Marginal model plots that showed the marginal response between the response and each predictor reproduce non-linear marginal relationships for the predictors (Figure 4). Although the mean function is a linear function of the predictors, the curvature in the smoothed plots is a result of non-linear mean functions among the predictors and does not indicate a faulty model [33]. On the contrary, the model, as represented by dashed lines in Figure 4, clearly matches the marginal relationships of the data represented by the solid lines.

**Table 3:** Analysis of deviance for acoustic tagged green sturgeon habitat model.

Model	Deviance Reduction	Residual df	Residual Deviance	F	Pr (>F)
Null			96.0		
Boulder	9.5	35	86.5	13.49	<0.01
Boulder^2^	45.4	34	41.2	64.71	<0.01
Rugosity^2^	2.0	33	39.1	2.89	0.099
Rugosity	17.3	32	21.9	24.62	<0.01

Model factors, proportion of boulders and rugosity, were chosen based on lowest qAIC from candidate factors shown in Table 2.

**Figure 5:**
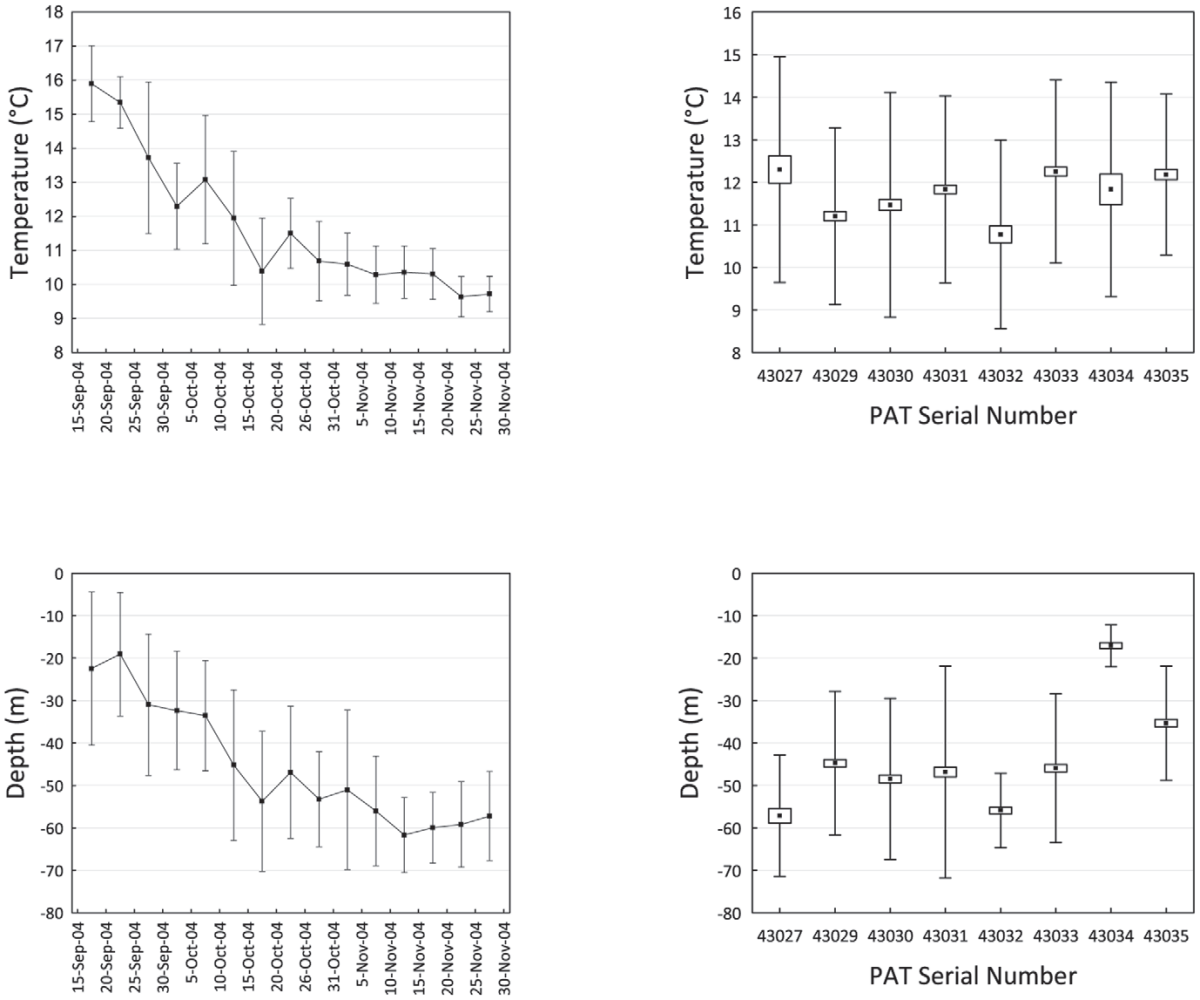
Temperature and Depth Summaries for PATs. Mean temperature and depth for eight green sturgeon fitted with pop-off archival tags (PAT) by date (left panels) and by individual sturgeon (right panels). Data points in the left panels indicate 5-day moving averages with standard deviations (whiskers). Data points in the right panels indicate means, bars denote 95% confidence intervals about the mean, and whiskers specify standard deviations.

Total lengths for eight green sturgeon fitted with PATs ranged from 158 to 222 cm. After leaving the Columbia and Rogue Rivers, sturgeon spent most time at depths from 20 to 60 m and at water temperatures from 16 to 9.5°C. Sturgeon moved progressively deeper throughout the study period until they reached a depth of about 60 m, which they tended to maintain for the remainder of the winter (Figure S1). Mean depths for PAT-fitted sturgeon were greater than 20 m after 20 September 2004 and depths were in the 50 to 60 m range from 15 October through 30 November 2004 (Figure 5). Mean temperatures experienced by individual sturgeon (12.3 to 10.8°C, SE = 0.07) were less variable than mean depths (17.1 to 57.1 m, SE = 0.47). Mean monthly ocean isothermal layer depth below sea surface (http://www.esrl.noaa.gov/psd, accessed 22 March 2011) during the study period indicates that sea temperatures were relatively uniform over the depths where the PAT fitted sturgeon were present. All PATs detached and downloaded inside of the 115 m depth contour from Coos Bay, Oregon, USA in the south to the northern tip of Vancouver Island, British Columbia, Canada in the north (Figure 1).

## Discussion

In many disciplines, making inferences by model selection is replacing the null hypothesis testing approach because it offers a robust framework for choosing from among multiple competing hypotheses without being restricted to evaluating the significance of a single model by an arbitrary probability threshold [32]. We used GLM selection to investigate the mean response of green sturgeon to components of seafloor physical habitat. GLMs are an extension of linear models that allow non-normal distributions of the response variable and have been used extensively in fisheries science [34]. Initially, we were naïve regarding the ecological relevance and appropriateness of the various seascape metrics available for inclusion in the model selection process. Nevertheless, we recognized that our measured seafloor components primarily characterized seafloor complexity in terms of variation in geographic relief and sediment size. Therefore, our implicit null hypothesis was that on average, green sturgeon utilized areas with seafloor complexity in equal proportion to that which was available. If the null hypothesis were true, then none of the variables should have had a strong relationship with sturgeon presence and the best models would still have weak predictive ability. The selected “best” GLM had both strong predictive ability and identified informative variables that suggested an optimal level of seafloor complexity for green sturgeon that was greater than the mean complexity available in the coastal study area. Our NMS analysis was consistent with GLM results and improved our understanding of the shape, strength, and direction of the habitat component gradients in the study area. By encapsulating the sturgeon site-duration gradient on a single axis or non-linear surface, it was straightforward to ascertain positive relationships with rugosity and other complexity surrogates, and a negative relationship with the proportion of sand substrate that is indicative of reduced seafloor complexity. Our statistical model of habitat use described the distribution of individual sturgeon across various habitat component gradients and provides a starting point for future habitat studies in which hypotheses regarding fine-scale habitat choices may be examined.

Because they are highly migratory, green sturgeon may experience environments ephemerally and choose to move to different locations depending on the timing of seasonal environmental variations. We detected most individuals for only a few days within the hydrophone array, indicating that they move extensively. Based on detachment locations of our PAT fitted sturgeon and previous studies, many, but not all, acoustically tagged sturgeon were likely migrating in a northward direction and moving deeper during our study period [4], [6]. It is evident that the behavior of green sturgeon regarding the depth they inhabit may vary greatly. For example, one individual (PAT serial number 43034) remained shallower than 20 m throughout the study period and did not migrate substantially while its PAT was attached. Detections recorded before mid-September must have been from sturgeon that were either residing in the coastal ocean or moving between spawning or summer estuarine holding areas and winter-feeding grounds. The period after mid-September is a period of ocean residence for all green sturgeon except for the very young that have not left their natal rivers and the remaining over-summering migrants from the previous spawning cycle.

The primary limitation of our study is that the spatial scale of our habitat analysis represents only a portion of the green sturgeon range, and late winter to early spring data are absent. Although we have no basis to presume that green sturgeon marine habitat preferences differ greatly during other times of year or in other locations, additional year-round, range wide data may corroborate our current results. In describing habitat utilized by sturgeon on an individual basis, summarized by weighted-averaging habitat values across all hydrophones, we essentially decomposed any spatial structure that may have existed in our dataset. Observations at geographic distances that are more or less similar to one another than expected by chance may increase errors and bias in GLMs [35], [36], [37]. However, the potential for spatial bias among detections for individuals is low because green sturgeon commonly travel daily distances that are many times farther than the length of our entire hydrophone array [4]. Furthermore, we analyzed averaged habitat values, rather than habitat values associated with individual detections.

Although differences in detection densities among physical habitat types should generally reflect differences in habitat quality, there are potential alternative and not mutually exclusive explanations for these differences [19], [38]. For example, sturgeon may spend more time in areas with high relief rock, boulders, and a complex ocean bottom because these features comprise navigational impediments. Yet, in this case, sturgeon should easily be able to avoid these rocky areas because this habitat is only intermittently distributed along the western coast of North America [39]. Regardless of the mechanism or cause of differences in habitat quality, models of habitat use overwhelmingly predict that there will be lower turnover rates among individuals occupying optimal habitats [40]. Therefore, our finding of a greater duration of sturgeon residence in areas with greater seafloor complexity likely indicates greater habitat quality such areas. Species characteristics that may decouple habitat quality and the density of individuals include: social dominance interactions, high reproductive capacity, and generalist habitat predilections [38]. But these characteristics tend to be less closely associated and less influential with large animals that occur at low densities such as green sturgeon, that also have no known social dominance interactions [38].

Anadromy in sturgeons is thought to be a secondary adaptation that facilitates exploitation of abundant benthic invertebrates in coastal marine habitats [41]. Fluctuating food resources in the coastal areas between spawning rivers and presumed rich feeding grounds to the north [4] could provide an explanation for observed differential migration, which has previously been observed in birds and fish [23], [42]. Most adult sturgeons fast continuously for several months and feed intensively for just a few months per year; for green sturgeon, feeding likely only occurs in marine or estuarine habitats [13], [41]. Prey availability appears to drive the marine distributions of Atlantic (*Acipenser oxyrinchus*) and Gulf (*Acipenser oxyrinchus desotoi*) sturgeon [43], [44]. Atlantic sturgeon have shown little or no habitat selection based on other factors, rather they only occur where they find prey [43]. Gulf sturgeon have been suggested to forage in a manner consistent with a Lévy search pattern in which they disperse in a random direction and continue until suitable prey patches are found [44], [45]. Other studies have suggested that Gulf sturgeon are distributed according to the presence of prey taxa, but they tend to have a highly structured spatial distribution wherein they pass over less favorable foraging areas directly toward preferred shallow sandy sites [46], [47], [48], [49]. European sturgeon (*Acipenser sturio*) have been reported over sandy areas as well, but they were also found in deeper waters over coarse, and sometimes rocky areas [16]. Green sturgeon disperse widely along the west coast, but in marine environments they may preferentially reside in areas that provide superior foraging opportunities such as in the estuarine environments at the mouths of large rivers or in the nearshore coastal ocean [4], [6], [28]. Because migratory behavior is likely an adaptation for exploiting seasonally available resources, sturgeon may spend more time in topographically complex areas in the coastal ocean when foraging among the soft sediments between rocky areas provides a substantial enough energetic advantage. However, there may be an additional pressure to forage in complex areas if it provides refuge from predators relative to open, soft bottom areas.

Predation or competition among other species may also influence sturgeon habitat preferences. For species such as the Gulf sturgeon, feeding only occurs during the winter months when there is the lowest predation potential from sharks and when there is less seasonal competition for food from teleost fishes [41]. Although almost nothing is known regarding green sturgeon natural predators in the ocean [50], one of the authors (STL) has observed injuries on green sturgeon captured in gill nets consistent with shark bites, and pinniped predation on sturgeon has also been reported [51], [52]. Green sturgeon may have evolved behaviors that reduce the risk of predation by creatures such as pinnipeds or sharks by avoiding detection while foraging in highly structured areas, and by migrating in a direction (overwintering at high latitudes) that contrasts with other temperate marine animals [4]. The decline of large, pelagic, predatory sharks and the subsequent increase in abundance of pinnipeds and demersal sharks could increase predation pressures and risk effects on green sturgeon, influencing feeding behavior, habitat use, distribution, and their associated food webs [53].

Our findings that green sturgeon are preferentially utilizing complex seafloor habitats gives rise to the possibility that although green sturgeon commonly occur at low densities in the coastal marine environment, specialized habitat requirements could have the potential to reduce green sturgeon fitness in suboptimal habitats and limit their geographic expansion [54]. Identification of green sturgeon physical habitat use patterns is a necessary step towards accurately defining the conditions that are essential for the survival of this species. We specifically addressed if green sturgeon occurrence differed among various substrate types and aspects of seafloor complexity. Our aim was to identify habitat components that best explained inter-habitat variability in green sturgeon distribution, and characterize features that define important coastal areas. Our statistical model, which identified a tendency for green sturgeon to be positively associated with complex benthic habitat during ocean residence, will provide valuable insight regarding the consequences of fisheries management actions, changes in marine environment conditions, and will eventually yield range-wide spatially explicit predictions of sturgeon distributions. Our analysis, in which we describe the average behavior of individuals across a stationary acoustic detection array, allowed us to characterize specific habitat components that are important to green sturgeon. We believe this is a novel approach that will lead to testable hypotheses concerning sturgeon foraging and predation avoidance, and may be readily applied to other comparable datasets.

## Materials and Methods

### Hydrophone arrays and habitat analysis

We conducted the hydrophone portion of the study at the Siletz Reef complex located off Lincoln City, OR (Figure 1). The site is characterized by extensive bedrock formations (0.75–200 ha), with stretches of high relief columnar structures and ridges interspersed with deep channels [55]. Water depth in the study area ranged from 20–69 m. In the summer of 2006, an array of moored Vemco VR2 and VR2W single channel acoustic receivers (Manufactured by Vemco®) was deployed at the Siletz Reef complex and at Government Point Reef, located 4 km south of Siletz Reef. The array was designed by Oregon Department of Fish and Wildlife to study acoustically tagged rockfish (*Sebastes spp.*) [56], but was also monitored for the presence of green sturgeon. Twenty receiver moorings were deployed on 15 June 2006 at Siletz Reef, three were deployed on Government Point Reef on 17 July 2006, and an additional eight were deployed 25 August and September 15 2006 within the existing Siletz array, to increase detection capability. The Siletz Reef grid encompassed 15 km^2^ and the Government Point grid covered 3.2 km^2^. Acoustic receivers were downloaded and removed on 26 October 2006.

We described habitat characteristics within a 250 m radius of the hydrophones using a two meter resolution bathymetric digital elevation model (DEM) and reclassified side-scan sonar data [57]. This radius for habitat description was chosen based on prior testing of acoustic detections within this grid (350–500 m detection range) [56], [58] which indicated that 250 m is a conservative distance for consistent valid acoustic detections. DEM derived variables included depth, aspect, rugosity and bathymetric position index (BPI), whereas reclassified side-scan sonar data quantified substrate types categorized as sand (0.06–2 mm dia., 40% of total area), high relief rock (>45° slope, 40% of total area), low relief rock (0–45° slope, 15% of total area), or boulder (0.25–3 m dia., 5% of total area). Cobble (64–250 mm dia., 0.01% of total area) and gravel (2–64 mm dia., 0.04% of total area) were also quantified as a part of the sonar image habitat interpretation, but occurred in negligible amounts within the region and were not included in the habitat analysis [57]. Aspect (i.e. cardinal direction of the pixel plane), which represents a proxy for exposure [59] was calculated using the Aspect tool in ArcToolbox (ESRI™ ArcMAP® v.10). The mode of aspect values within the buffer was transformed to the degree of eastness to account for circularity in this 360 degree directional measurement [60]. We calculated both rugosity and BPI using the Benthic Terrain Modeler extension (ESRI™ ArcMAP® v.10) [26], [61]. We calculated rugosity, a measure of the relative relief of an area, as the ratio of the area represented by a pixel to the planar area of a complex surface described by the relative elevations of the eight immediately neighboring pixels [29]. BPI is a scale dependent measure of the relative position of a location with regard to the surrounding topology. We calculated it as the ratio of elevations to that of the mean elevation within a given annulus of the location. BPI value grids were standardized to control for scale dependence and reclassified to produce a raster of categorical topographic positions [62]. We calculated the proportional area of six BPI classifications within a 250 m buffer of each hydrophone, these included: “peak/ridge,” “upper slope,” “middle slope,” “flat/plain,” “lower slope,” and “valley/crevice.” All proportional values were subsequently arcsine square root transformed prior to statistical analysis.

### Fish tagging

Thirty-seven adult green sturgeon were captured by various researchers in spawning rivers, bays, and estuaries with gill nets or by angling and were acoustic tagged and released from August 2003 to October 2005 (Table 1). Coded ultrasonic pinger tags (Vemco V16-6H) with a tag life of 3–5 years were implanted surgically into the abdominal cavity. An additional eight green sturgeon (Table 1) were captured in the mouth of the Columbia River (n = 7) or Rogue River (n = 1) in July and August 2004 and externally fitted with PATs (Microwave Telemetry® model PTT-100) that recorded temperature and depth at one hour intervals. The PATs released, ascended to the ocean surface and transmitted data to NOAA satellites from January to December 2005. We summarized temperature and depth data from September to December 2004 for comparison with the acoustic tag data set during a period in which all of the PAT fitted fish were consistently recorded in a marine environment. Specific fish tagging and handling details are given by Erickson and Webb (2007), Kelly et al. (2007), Moser and Lindley (2007) and Lindley et al. (2008) for acoustic tags [4], [7], [12], [13], and are given by Erickson and Hightower (2007) for PATs [6].

### Data Analysis

We quantified the cumulative number of days that tagged sturgeon were present in the vicinity of hydrophones by summing the time elapsed between detections at each hydrophone in which at least two sequential detections occurred with no intervening detections at another hydrophone. We objectively described the relationship among the 37 acoustically tagged sturgeon and the hydrophone locations using NMS [63], [64] implemented with PC-ORD software [65]. We positioned individual fish (rows) by average Bray-Curtis dissimilarity distances [66] according to covariation and association among cumulative duration of individual sturgeon presence at the hydrophones (columns). We began with a random initial starting configuration and determined the appropriate number of dimensions for the best ordination by examining stress versus dimension plots. We determined the best solution when the standard deviation in stress over the preceding 10 iterations reached 0.00001. We rotated our final NMS plot so that the horizontal axis represented the direction of the maximum linear correlation of sturgeon detection-days with the NMS plot scores. We performed an indirect gradient analysis of the relationship among the habitat variables and the duration of sturgeon presence by representing each habitat variable as a contour gradient overlaid on top of the previously constructed NMS ordination plot [67], [68] using R software [69], [70]. We calculated habitat variable ordination scores by weighted averaging site score values for each variable. We constructed habitat variable contours with non-parametrically smoothed surfaces that were fitted from general additive models (GAM, Gaussian error distribution with identity link) with thin plate splines [71]. The degree of smoothing was determined using cross-validated r^2^ to determine goodness-of-fit. We tested the significance of each contour surface with an ANOVA in GAM. We also constructed a similar contour plot using NMS site ordination scores with contours based on sturgeon detection-days at each site. We calculated coefficients of determination (R^2^) for NMS horizontal axis scores (linear) and for fitted contours (non-linear).

We then calculated the average proportion of time that fish spent associated with each habitat type by weighting the habitat values within each hydrophone buffer by its proportion of the total duration at all hydrophones to produce a weighted average for each habitat type for each fish. This calculation for a given habitat variable, 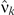 is:
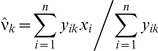
where *x* is the environmental variable, *x_i_* is the value of *x* at hydrophone *i*, and *y_ik_* is the amount of time spent by individual *k* at hydrophone *i*. This procedure generated a set of average habitat component values unique to each fish that we used to construct GLMs [72] of physical habitat use with a quasi-Poisson error distribution to allow for overdispersion in inferences and a log link [73], [74]. Our response variable was the total duration in days that we detected a tagged fish at all hydrophones (detection-days). We chose candidate variables for the GLMs by visually examining plots of all variables against one another and the response variable to identify plausible relationships between species and environmental variables. We incorporated quadratic variable counterparts in the GLM process for candidate variables with a theoretical rationale (i.e. a hypothetical optimal value) indicating a nonlinear relationship (i.e. depth, rugosity, sand, low relief rock, high relief rock, boulders) with the response variable, resulting in 19 total candidate variables. We selected GLMs based on lowest qAIC values using R (Version 2.12.1) software [69] and the MuMIn package [75]. Because each of the variables that we selected had a biologically realistic basis for being included and there was no rational justification for excluding more variables, we constructed all subsets of potential models, but we allowed a maximum of four variables in any given model to help avoid model overfitting. We used the variance inflation factor to measure possible collinearity among explanatory variables, where a value >10 was considered indicative of collinearity [76]. We calculated jackknifed R^2^ and RMSE values for the best models and constructed marginal model plots of predictor variables using the car package in R [33], [77] to evaluate model fit. In both the GLM and the NMS analyses, we accounted for the fact that receivers were deployed for variable lengths of time by dividing the total number of detection-days for each receiver by the number of days the receiver was deployed.

We calculated 5-day moving averages for temperature and depth for seven PAT monitored sturgeon from the 15 September to 30 November 2004. This time period was summarized to exhibit typical temperatures and depths that sturgeon experience upon returning to the open ocean from a summer spent in bays or estuaries (mid-September to mid-October) and conditions in the open ocean (mid-October to mid-November).

## Supporting Information

Figure S1 Temperature (top panel) and depth (bottom panel) recorded by pop-off archival tags for green sturgeon in this study from January 2004 to January 2005.
(TIF)
