# Supplementary figures and images for: Green Sturgeon Physical Habitat Use in the Coastal Pacific Ocean
Source: PLoS One. 2011 Sep 22;6(9):e25156. doi: 10.1371/journal.pone.0025156 (PMC3178618; doi:10.1371/journal.pone.0025156)

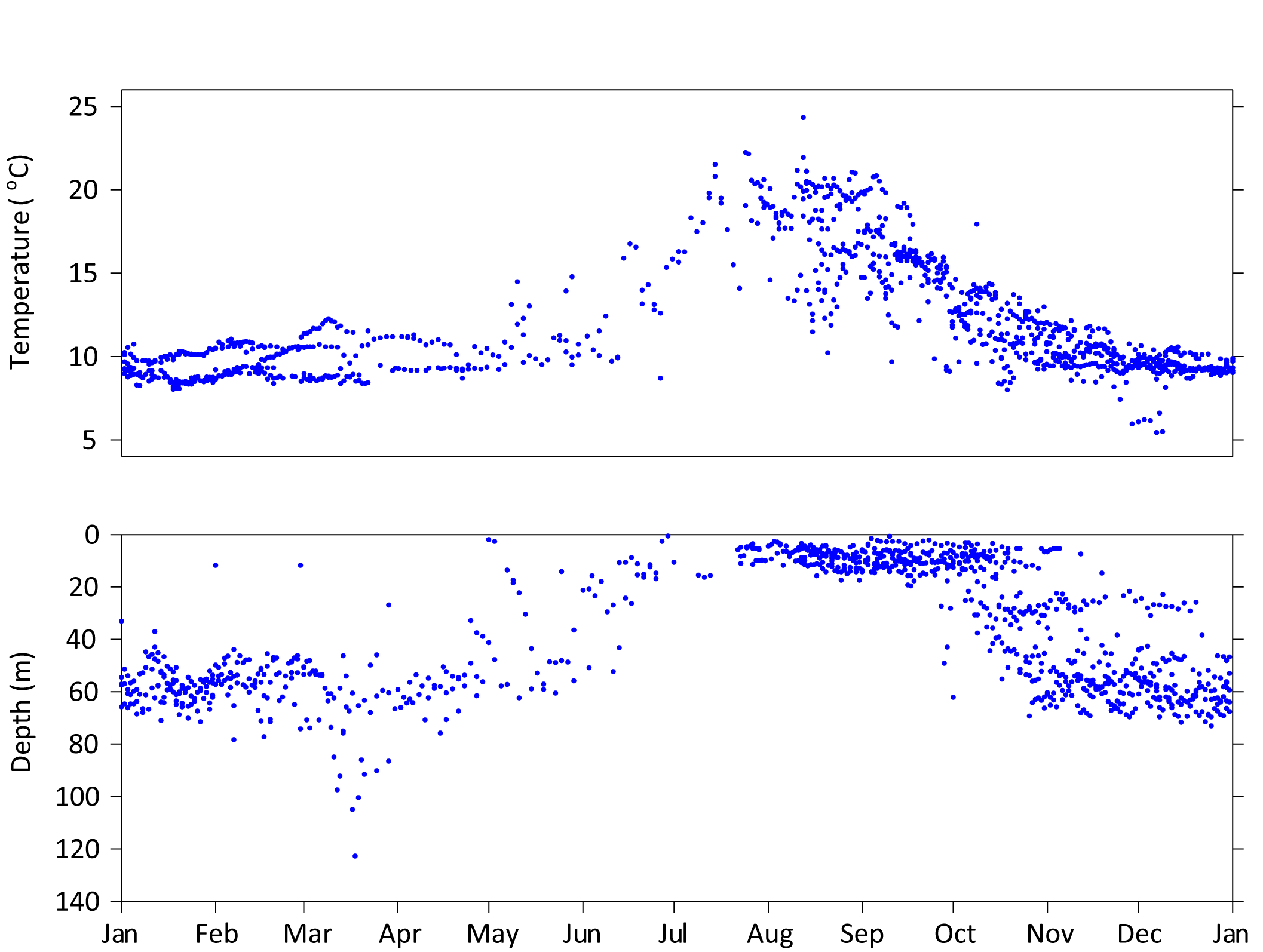

Supplement: Figure S1 — Temperature (top panel) and depth (bottom panel) recorded by pop-off archival tags for green sturgeon in this study from January 2004 to January 2005. (TIF) [file pone.0025156.s001.tif]
